# Psychological distress and coping strategies among the caretakers of children with transfusion-dependent thalassemia

**DOI:** 10.3389/fped.2022.941202

**Published:** 2022-08-22

**Authors:** Azizah Othman, Mohd Sufian Ardi Abdul Ghani, Fahisham Taib, Norsarwany Mohamad

**Affiliations:** ^1^Department of Paediatrics, School of Medical Sciences, Universiti Sains Malaysia, Kota Bharu, Malaysia; ^2^Paediatrics Clinic, Hospital Universiti Sains Malaysia, Kota Bharu, Malaysia

**Keywords:** psychological distress, coping, children, caretakers, thalassemia, chronic illness

## Abstract

**Introduction:**

Thalassemia is a chronic childhood disease that could result in psychological distress not only to the patients but also to their caretakers. Caretakers utilize different coping strategies to reduce stress and maintain a good quality of life.

**Objective:**

The study aims to measure the level of psychological distress among caretakers of transfusion-dependent thalassemia patients and identify coping strategies used by them, as well as examine factors related to both outcome measures.

**Methods:**

Sixty-eight (*N* = 68) caretakers of children with transfusion-dependent thalassemia agreed to participate in the study when they were approached during their visits to one of three major hospitals in Kelantan, Malaysia, for the children's medical treatment. They completed the Malay validated Depression Anxiety and Stress Scale 21 (DASS 21) and Brief-COPE self-report, in addition to a brief study proforma.

**Results:**

The majority of the participants reported feeling psychologically well, with no related scores in depression, anxiety, and stress sub-scales. The mean score for anxiety and stress sub-scales were 3.54 (SD = 3.54) and 4.25 (SD = 3.26) respectively. The median score for the depression sub-scale was 2.00 (IQR 4.00). The three mostly utilized coping strategies were religion, acceptance, and positive reframing. Those with depressed and anxious moods were found to engage more in negative coping strategies including substance abuse, denial, and behavioral disengagement. Being female, of younger age, employed, with higher educational level, and income status was found to significantly influence the adoption of positive reframing as a coping strategy.

**Conclusion:**

Psychological distress such as elevated anxiety and depression was found among a small portion of caretakers who have children with thalassemia whose treatment required blood transfusion. They were noted to apply more maladaptive coping strategies compared to their psychologically well counterparts.

## Introduction

Thalassemia is an autosomal recessive disorder of red blood cells associated with the defect in the synthesis of normal hemoglobin. Around 900,000 births of clinically significant thalassemia disorders are estimated to occur in the next 20 years (1). Thalassemia is considered one of the most common genetic diseases in the world, affecting thalassemic belt countries such as the Mediterranean, Asia Minor, and Southeast Asia, including Malaysia. According to the Malaysian Thalassemia Registry, there are 4,759 patients diagnosed with thalassemia who are also registered as transfusion-dependent type (2). It was shown that 6.8% of Malaysians are carriers of the beta thalassemia trait with the incidence predominantly seen in Malay and Chinese ethnicity. Malaysia's thalassemic population cohort has shown a right shift from the presence of many surviving and older patients. These patients are largely a transfusion-dependent thalassemia (TDT) group and would potentially possess iron toxicity-related diseases.

Chronic illness such as thalassemia often poses negative effects on the psychological functioning of patients and their families as high as 1.5 to 3 times compared to their healthy peers (3). When thalassemia affects the children, it is not only a burden to the children but also to their parents and the whole family (4). A qualitative study on Malaysian patients and their parents have highlighted concerns and adverse impacts related to poor school performance, low self-image, limited employment opportunity, marriage, and financial difficulty as well as challenges to integrating into society (4).

A study on 150 parents of children with a β-thalassemia major in India indicated adjustment disorder (10%), depressive disorder (33%), anxiety disorder (10%), and somatoform disorder (11%) among the caretakers (5). Frequent hospitalization, delayed disease presentation, and school-going age patients were associated with higher psychological problems among the families (6).

The study investigated the level of psychological distress and the types of coping utilized by caretakers of TDT children. It sought to examine if there were any differences between those with and without distress about coping strategies they used, as well as the predictors of utilization of positive coping strategies.

## Method

### Study design and location

A cross-sectional study was conducted in three hospitals in east-coast Malaysia that serve as referral treatment centers managing complex thalassemia cases in that region namely Hospital Universiti Sains Malaysia (Hospital USM), Hospital Raja Perempuan Zainab II (HRPZ II) Kota Bharu, and Hospital Kuala Krai (HKK). Hospital USM is a tertiary center for teaching and pediatric referral cases with a capacity of 723 beds. HRPZ II is the biggest government hospital located in the heart of Kota Bharu, the capital state of Kelantan with 937 beds capacity. It has a multi-disciplinary team covering the population from all parts of the state. Meanwhile, HKK is a secondary hospital approximately 60 miles away from the capital of Kota Bharu with 268 beds.

### Participants

In this study, the caretaker is defined as the main person who takes care of the patient, most of the time. They are mainly one of the parents—mother or father or any other person who plays a major role in taking care of the child, during the study period, such as a grandmother or aunt.

Caretakers of TDT children from any of the above hospitals, including those whose children were recently diagnosed, were invited to participate in this study. Caretakers who were cognitively incompetent, known to have serious medical or psychiatric illnesses, or refused to consent or participate were excluded from the study.

Psychological distress is characterized by identical combinations of symptoms ranging from stress, depression, and general anxiety symptoms that may result in functional disabilities and behavioral problems. Coping is defined as behavioral and cognitive efforts made by individuals in attempting to deal with stressful situations.

### Measures

Depression Anxiety and Stress Scale 21 (DASS 21) was used to measure caretakers' psychological stress levels. This self-report scale consists of three sub-scales with seven items each and is designed to provide measures of the negative affective states of depression, anxiety, and stress. The depression sub-scale assesses dysphoria, hopelessness, self-criticism, lack of interest or involvement in daily activities, anhedonia, and inertia. In the anxiety sub-scale, autonomic arousal, skeletal muscle effects, situational anxiety, and subjective experience of anxious affect are assessed. The stress sub-scale assesses difficulty relaxing, nervous arousal, being easily upset or agitated, irritable or over-reactive, and impatient (7).

The score is presented as a total score and a score for the three subscales. The DASS 21 is a quantitative screening of distress and not a categorical measure of clinical diagnoses. Yet for clinical reasons, it is beneficial to categorize the degree of severity relative to the population. The cut-off scores have been developed for defining normal, mild, moderate, severe, and extremely severe scores for each DASS sub-scale as below in Table 1 (7).

**Table 1:** The DASS 21 recommended cut-off points (7).

	**Depression**	**Anxiety**	**Stress**
Mild	0–9	0–7	0–14
Normal	10–13	8–9	15–18
Moderate	14–20	10–14	19–25
Severe	21–27	15–19	26–33
Extremely Severe	28+	20+	34+

The Malay version of DASS 21 was used in this study. It has been validated by Ramli Musa et al. (8) and Cronbach's alpha value for overall items was 0.90 (9).

Brief-COPE was utilized to identify the coping behavior among adults. It was originally developed by Carver in 1997. In Malaysia, the Brief-COPE scale has been validated in 2009 by N.Yusoff, et al. with internal consistency values ranging from 0.5–0.99 (10). The scale comprises 28 items and is categorized into 14 subscales. These represent coping strategies such as self-distraction, active coping, denial, substance use, use of emotional support, use of instrumental support, behavioral disengagement, venting, positive reframing, planning, humor, acceptance, religion, and self-blame. The scale is rated by the four-point Likert scale, ranging from “I haven't been doing this at all” (score one) to “I have been doing this a lot” (score four). The total possible scores for all 28 items are 112, yet for each sub-scale, the total potential score is 8. A higher score represents greater coping strategies used by the participants.

A standard proforma gathered participants' demographic data such as age, gender, ethnicity, occupation, educational background, and family-related information.

### Data collection

Caretakers who accompanied their children's treatment and follow-up at the outpatient clinic were approached and invited to participate in the study. An explanation of the study and its potential benefits was provided by the main researcher. Following consent, they were requested to fill up the DASS 21 and Brief-COPE questionnaires, as well as the study proforma. Whilst the potential participants might have felt obliged to participate in this study, they were guaranteed that their participation is on a voluntary basis, and their child's treatment will not be jeopardized despite their refusal to participate.

### Statistical analysis

Data were analyzed using SPSS version 20. Descriptive statistics were performed on sociodemographic variables. For categorized data, frequency and percentages (%) were presented in the result. For numerical data, mean and standard deviation (SD) were used if the variables were normally distributed and median and inter-quartile range (IQR) for skewed variables. For univariate analysis, the independent *t*-test or Mann-Whitney test were executed for the 2 groups' mean comparison. Multiple Logistic Regression was used to estimate the associated factors affecting DASS 21 and coping strategies. The study outcome was a dichotomous binary categorical variable for both DASS 21 and brief COPE.

## Results

### Socio-demographic characteristics

There were 68 caretakers who voluntarily agreed to take part in this study. More than half of the participants were female [55.9% (*n* = 38)], while the male caretakers accounted for 44.1% (*n* = 30). A majority of them were aged between 40 and 60 years and belonged to the middle adulthood age group 64.7% (*n* = 44); the rest 35.3% (*n* = 24) were in the younger adult age group aged between 20 and 39 years. Almost all of them are Malay and Muslim [98.5% (*n* = 67]. A majority of the caretakers had completed secondary schooling, which is approximately twelve years of undergoing formal educational experience (*n* = 53). Most of them were employed 60.3% (*n* = 41) during the data collection period. From a socioeconomic perspective, middle- and high-income groups outnumbered the low-income group by 38.2%. Significantly, 14 (20.6%) caretakers reported having two or more TDT children in their house to be managed. Participants' socio-demographic information is presented in Table 2.

**Table 2:** Sociodemographic characteristics of the participants (*N* = 68).

**Characteristics**	***n* (%)**
**Hospital**	
Hospital USM HRPZ II HKK	14 (20.6) 48 (70.6) 6 (8.8)
**Age**	
Young adult (Age ranges: 20–39) Middle age (Age range: 40–60)	24 (35.3) 44 (64.7)
**Gender**	
Male Female	30 (44.1) 38 (55.9)
**Race**	
Malay Chinese	67 (98.5) 1 (1.5)
**Number of children with thalassemia in the family**	
One only Two or more	54 (79.4) 14 (20.6)
**Employment status**	
Employed Unemployed	41 (60.3) 27 (39.7)
**Socioeconomic status**	
Low [B40] Middle and High [M40 and T20]	21 (30.9) 47 (69.1)
**Highest level of education**	
Tertiary Secondary or below	15 (22.1) 53 (73.9)

### Prevalence of psychological distress

The prevalence of depression, anxiety, and stress symptoms among the caretakers of TDT children was estimated using DASS 21 (refer Table 3). The majority of the participants reported feeling psychologically well. Sixty-three (92.6%) of them were not anxious, 64 (94.1%) were not depressed, and none reported any significant stress. The mean for anxiety and stress symptoms were 3.54 (SD = 3.54) and 4.25 (SD = 3.26) respectively. Due to the skewed data distribution for the Depression sub-scale, the median and the interquartile range are reported. The median score for depressive symptoms was 2.00 (IQR 4.00).

**Table 3:** The frequency and percentage of participants reported experiencing normal, mild, moderate, and severe levels of depressive, anxiety, and stress symptoms based on DASS 21 (*N* = 68).

**Category/**	**Normal**	**Mild**	**Moderate**	**Severe**
**Subscale**	***n* (%)**	***n* (%)**	***n* (%)**	***n* (%)**
Depression	64 (94.1)	2 (2.9)	2 (2.9)	–
Anxiety	63 (92.6)	1 (1.5)	3 (4.4)	1 (1.5)
Stress	68 (100)	–	–	–

There were four (5.9%) caretakers who reported experiencing mild to moderate depressive symptoms and five (7.4%) of them also experienced mild to severe anxiety symptoms. None of them reported experiencing significant stress symptoms.

### Types of coping strategies utilized

Through the Brief-COPE scale, we presented the means and standard deviations for each coping strategy when the data were normally distributed, and median and interquartile ranges, when the data were not normally distributed, in Table 4. We identified the most commonly utilized coping strategies by caretakers of TDT children was “religion” with a median score of 7.00 (IQR = 2.00). This was followed by “acceptance” and “positive reframing” with a median score of 6.00 (IQR = 3.00) and a mean score of 5.81 (SD = 1.80) respectively.

**Table 4:** The mean and standard deviation (SD) or the median and interquartile range (IQR) for each type of coping strategy utilized by the participants as reported in Brief–COPE (*N* = 68).

**Item**	**Mean (SD)** **Median (IQR)^∧^**
Religion	7.00 (2.00)^∧^
Acceptance	6.00 (3.00)^∧^
Positive reframing	5.81 (1.80)
Active coping	5.49 (1.82)
Planning	5.25 (1.69)
Use of instrumental support	4.87 (1.62)
Use of emotional support	4.56 (1.54)
Self– distraction	4.53 (1.69)
Venting	4.18 (1.64)
Humor	3.94 (1.35)
Denial	3.29 (1.52)
Substance use	2.00 (0.00)^∧^
Self– blame	2.00 (2.00)^∧^
Behavior disengagement	2.00 (1.00)^∧^

^∧^ Median (IQR) due to skewed data.

We compared the scores of groups of participants who reported experiencing depression and anxiety to those who reported normal functioning, with every type of coping strategy they utilized. The results are presented in Table 5.

**Table 5:** The median and Interquartile Range (IQR) scores of different coping strategies utilized by participants who reported normal psychological functioning compared to those who reported having depressive and anxiety symptoms.

	**Median** **(IQR)**		**p**	**Median** **(IQR)**	**p**
**Coping strategies**	**Normal**	**Depression**		**Anxiety**	
Substance use	2.00 (0.00)	4.50 (3.00)	<0.01*	4.00 (4.00)	<0.01*
Denial	3.00 (2.00)	5.50 (3.00)	0.06	5.50 (3.00)	0.02*
Behavior disengagement	2.00 (0.00)	3.00 (4.00)	0.14	4.00 (3.00)	0.03*
Self–distraction	5.00 (3.00)	0.50 (3.00)	0.13	3.00 (3.00)	0.13
Active coping	6.00 (3.00)	4.50 (2.00)	0.31	5.00 (2.00)	0.40
Emotional support	4.56 (1.56)^∧^	4.50 (1.29)^∧^	0.94	4.50 (1.29)^∧^	0.94
Instrumental support	5.00 (2.00)	6.00 (2.00)	0.47	6.00 (2.00)	0.50
Venting	4.17 (1.65)^∧^	4.25 (1.71)^∧^	0.93	5.00(3.00)^∧^	0.45
Positive reframing	6.00 (2.00)	6.00 (2.00)	0.43	6.00 (3.00)	0.18
Planning	5.28 (1.71)^∧^	4.75 (1.26)^∧^	0.55	5.00 (2.00)^∧^	0.72
Humor	4.00 (2.00)	4.50 (2.00)	0.22	4.00 (2.00)	0.28
Acceptance	6.00 (3.00)	6.00 (2.00)	0.59	6.00 (1.00)	0.54
Religion	7.00 (2.00)	6.50 (4.00)	0.45	6.00 (3.00)	0.29
Self–blame	2.00 (2.00)	4.00 (4.00)	0.15	4.00 (3.00)	0.07

^∧^Mean (SD) for normally distributed data.

^*^p < 0.05.

There was a significant difference in substance abuse as a coping strategy among those with depression and anxiety (*p* < 0.01), as compared to those who reported normal psychological functioning.

Similarly, we found a significant difference in the use of denial as a coping strategy among participants with anxiety (*p* = 0.02), and depression (*p* = 0.06), compared to others who reported normal psychological functioning.

Finally, we noted that those with anxiety displayed more behavioral disengagement compared to caretakers who reported normal psychological functioning (*p* = 0.03).

In short, those with psychological distress reported more use of substances, denial, and behavioral disengagement, as compared to those having normal psychological functioning.

Using multiple logistic regression, we estimated the factors associated with depressive, anxious, and stress symptoms as well as coping strategies used by caretakers of children with transfusion-dependent thalassemia. The association between positive reframing and demographic data was noted to be statistically significant where middle-aged caretakers utilized positive reframing as a coping strategy 0.23 times less compared to younger adult caretakers. Similarly, female caretakers utilized positive reframing 13.54 times more compared to male caretakers. Those with comparatively lower levels of formal education used positive reframing 0.08 times less than caretakers with higher levels of education. The results are presented in Table 6. No other significant results were found with other variables of interest.

**Table 6:** Factors associated with the use of positive reframing as a coping strategy by the simple and multiple logistic regression model.

**Variable**	**Simple logistic regression**			**Multiple logistic regression**ª		
	**b**	**Crude OR (95% CI)**	**p**	**b**	**Crude OR (95% CI)**	**p**
**Age Group**						
Young adult	0	1		0	1	
Middle age	−1.48	0.23 (0.06,0.87)	0.03	−1.48	0.23 (0.06,0.87)	0.03
**Gender**						
Male	0	1		0	1	
Female	2.61	13.54(1.43, 128.25)	0.02	2.61	13.54(1.43, 128.25)	0.02
**Highest Level of Education**						
Tertiary	0	1		0	1	
Secondary and below	−2.25	0.11(0.01,0.89)	0.04	−2.52	0.08(0.01,0.79)	0.03
**Employment status**						
Employed	0	1		0	1	
Unemployed	−2.38	0.09 (0.01, 0.84)	0.03	−2.38	0.09 (0.01, 0.84)	0.03

ªBackward LR Multiple Logistic Regression model was applied, Multicollinearity and interaction terms were checked and not found, Hosmer–Lemeshow test, (p = 1.00), classification table (overall correctly classified percentage = 75%), and area under the ROC curve (20%) were applied to check the model fitness.

## Discussion

There are not many studies that focus on psychological distress levels among caretakers of children with thalassemia who are transfusion dependent and their coping strategies in Malaysia. Previous findings showed that parents experienced psychological distress and they used positive religious coping methods more often than negative religious coping methods (11). Another study utilized the same measures as in this present study and documented psychological distress in 67.5% of caretakers (12).

Given this premise, we attempted to analyze psychological distress and coping strategies among caretakers of children with transfusion-dependent thalassemia, as well as to explore psychosocial associated factors related to the outcome variables. As a chronic, hereditary disease, thalassemia places the patients and their families under a great deal of stress which could directly affect their quality of life. When the children are dependent on regular, lifetime blood transfusion to survive, the challenges for them, as well as the caregivers, are presumed to be much more difficult.

The prevalence of psychological distress in our study was 13.2 % (*n* = 9). Specifically, 7.4% of the caretakers had mild to severe anxiety and 5.9% of them had mild to moderate depressive symptoms. Stress in caregivers can be attributed to events such as frequent treatment procedures, hospital visits, expected complications, poor life expectancy, and monetary burden to them.

There were some disturbing findings when we linked the caretakers' distress and coping strategies. Caretakers with depressed moods reported more substance abuse, whilst those with anxiety were in denial and behaviorally disconnected from their surroundings to cope, more significantly, in comparison to psychologically well caretakers. Kasi et al. (13) grouped these three types of coping strategies as maladaptive (13). Considering all caretakers were free from any psychiatric illnesses during data collection, the possible explanation for this result could be likely personality determinants. The five-factor model of personality is currently accepted as a viable explanation of normal adult personality functioning (14). One of these factors, neuroticism, is said to be related to stress and coping (15) and may be related to substance use (16). Neuroticism involves negative states such as anxiety, depression, hostility, self-consciousness, impulsiveness, and vulnerability (14). Therefore, persons high in neuroticism generally use maladaptive coping strategies.

However, no significant stress symptom was reported by the caretakers in this study. Henry et al. (17) assessed whether stress, as indexed by DASS 21, is synonymous with negative affectivity or whether it represents a related, but distinct, construct. The researchers concluded that although the three DASS 21 scales index are a substantial common factor (i.e., general psychological distress), they also contain variance that is specific to each scale (17). The correlational analyses support that the stress scale represents a legitimate measure and is not simply equivalent to negative affectivity. Therefore, our finding that stress was not elated in our sampling is indeed acceptable (18).

Correspondingly, the percentage of psychological distress in our study was five times lesser than in previous studies (12). This finding could likely be explained by effective coping strategies the caretakers have utilized. To reiterate, the three most popular coping strategies were religion, acceptance, and positive reframing. These results are consistent with previous studies (19). Religious coping promotes relevant cognitive coping strategies such as acceptance, hope, positive thinking, as well as making meaning out of stressful times, which is similar to cognitive restructuring, an evident-based effective stress management technique. This may explain the low incidence of psychological distress among the participants in this group.

Previous studies have examined religious coping in the context of chronic poverty-related stress and suggested that religiosity can buffer against the negative effects of stress. Religiosity and religious coping likely serve several functions to protect one against stress. Being a believer who is convinced that everything in this world is best planned by the Almighty God provides a sense of hopefulness that is comforting. For those who are highly involved in religious communities, religiosity provides social support and a place to get validation, respect, and a sense of acceptance (19). These factors may have similarly promoted healthy psychological functioning among the caretakers.

Financial status had been established to be associated with psychological distress. Whilst epidemiologic studies confirm that low-income populations are the groups most vulnerable to mental health problems, interestingly it is not the case in the present study. This could be due to good social support which protects against chronic stress (20). The extended family unit is an integral characteristic in Kelantan, Malaysia, and thus social support is accessible *via* family interdependency and close connections. Living in a predominantly rural area, unlike urban settlements, the ecology provides for low-cost shelters, abundant natural resources, and kin-dependent social support networks (21) which help buffer psychological stress.

Several other predictors namely age, gender, employment status, education level, and the number of dependencies were further examined. However, they were not associated with psychological distress, or specific coping strategies, except for positive reframing.

About 93% of female caretakers in this study adopted positive reframing as a coping strategy. Findings on studies of gender differences in coping behavior are not definitive. Previous studies found women were more likely to use emotion-focused coping (22). Emotion-focused coping may involve the use of behavioral and/or cognitive strategies such as receiving emotional support from friends and family and positive reframing. It can be active or avoidant emotion-focused coping. Active emotion-focused coping such as positive reframing is generally viewed as being an adaptive emotion regulation strategy whereas avoidant emotion-focused coping such as self-distraction where one tries to avoid the stressor is seen to be maladaptive (23). Since positive reframing is under the umbrella of emotion-focused coping, this present finding is comparable.

One study pertaining to age and coping among a European-American sample suggested that most individuals showed more adaptive and less maladaptive coping and defense strategies from adolescence until middle age or later (24). This study found that young adults coped more with humor (88%) and positive reframing (81%). Humor could be an adaptive or maladaptive coping style, but Brief-COPE would not be able to differentiate between the two coping styles.

Socioeconomic factors such as level of education had substantial effects on coping strategies in both genders and there was a positive relationship between higher educational levels and adaptive coping strategies and a negative relationship between lower educational levels and maladaptive coping strategies (25). Consistently, caretakers with a higher level of education in this study coped with positive reframing (93%) more than their counterparts.

Being employed was also linked with adaptive coping, which itself was a self-distraction and positive reframing coping style. Employment is generally the most important means to obtain adequate economic resources and is essential for material well-being. Work is central to an individual's identity, social roles, and social status. Employment and socio-economic status are the main drivers of social gradients in physical and mental health and mortality (26). Employed caretakers coped using positive reframing (92%) more than other counterparts, meanwhile, self-distraction was (87%) more than unemployed caretakers.

Understanding the psychological implications among caretakers resulted in many undesirable effects which would implicate them as the invisible patients themselves. This has highlighted the need to ensure a balanced approach in managing patients and their caretakers throughout the treatment of the disease. This can be favorably improved through identifying and frequent screening of caretakers' psychological impact throughout the disease trajectory. Future studies should explore interventions that would minimize the stress of caregiving to children with chronic illnesses such as thalassemia.

## Limitations and further recommendations

The study is limited by a relatively small sample size which could affect the statistical analysis and hence the study outcomes. It may be also confounded by single predominant Malay race sampling, acquired from a single state location in Malaysia, which may somehow subscribe to homogenous coping responses in dealing with difficult situations, such as having TDT children, thus generalization of the findings should be made with caution.

## Conclusion

Psychological distress was found among a small portion of caretakers who have children with thalassemia whose treatment required blood transfusion. We noted there was an incidence of anxiety and depressive symptoms, although not as high as in other studies, with alarming findings related to substance abuse, denial, and behavioral disengagement as coping strategies. Yet, the majority of the caretakers had utilized religion, acceptance, and positive reframing as coping methods to deal with the psychological distress while managing the TDT children and were reported to be psychologically stable.

## Data availability statement

The original contributions presented in the study are included in the article/supplementary material, further inquiries can be directed to the corresponding author.

## Ethics statement

The study protocol was approved by the Research and Ethics Committee, School of Medical Sciences, Universiti Sains Malaysia (USM/JEPeM/[268.4(1.6)]) and the Medical Research and Ethics Committee, Ministry of Health, Malaysia (NMRR -13-910-17085[IIR]).

## Author contributions

AO designed the study, revised the analyses, and prepared the final manuscript in consultation with FT and NM. MA collected and analyzed the data and wrote the first manuscript draft. FT polished the manuscript and made required revision. NM planned and supervised the project and overall research implementation. All authors contributed to the final manuscript.

## Funding

This project was funded by a Research University Individual Grant Universiti Sains Malaysia 1001/PPSP/8012292.

## Conflict of interest

The authors declare that the research was conducted in the absence of any commercial or financial relationships that could be construed as a potential conflict of interest.

## Publisher's note

All claims expressed in this article are solely those of the authors and do not necessarily represent those of their affiliated organizations, or those of the publisher, the editors and the reviewers. Any product that may be evaluated in this article, or claim that may be made by its manufacturer, is not guaranteed or endorsed by the publisher.
